# On the Burr XII-moment exponential distribution

**DOI:** 10.1371/journal.pone.0246935

**Published:** 2021-02-22

**Authors:** Fiaz Ahmad Bhatti, G. G. Hamedani, Mustafa Ç. Korkmaz, Wenhui Sheng, Azeem Ali

**Affiliations:** 1 National College of Business Administration and Economics, Lahore, Pakistan; 2 Marquette University, Milwaukee, WI, United States of America; 3 Department of Measurement and Evaluation, Artvin Çoruh University, Artvin, Turkey; 4 University of Veterinary and Animal Sciences, Lahore, Pakistan; Tongii University, CHINA

## Abstract

In this study, a new flexible lifetime model called Burr XII moment exponential (BXII-ME) distribution is introduced. We derive some of its mathematical properties including the ordinary moments, conditional moments, reliability measures and characterizations. We employ different estimation methods such as the maximum likelihood, maximum product spacings, least squares, weighted least squares, Cramer-von Mises and Anderson-Darling methods for estimating the model parameters. We perform simulation studies on the basis of the graphical results to see the performance of the above estimators of the BXII-ME distribution. We verify the potentiality of the BXII-ME model via monthly actual taxes revenue and fatigue life applications.

## 1. Introduction

Data analysis is imperious in every aspect of statistical analysis. The statistical characteristics such as skewness, kurtosis, bimodality, monotonic and non-monotonic failure rates are obtained from datasets. The selection of a suitable model for data analysis is a challenging task because it depends on the nature of the dataset. However, if a wrong model is applied to analyze the dataset it leads to loss of information and invalid inferences. It is obligatory to identify the most suitable model for the given dataset.

In the recent decade, many continuous distributions have been introduced in the statistical literature. Some of these distributions, however, are not flexible enough for data sets from survival analysis, life testing, reliability, finance, environmental sciences, biometry, hydrology, ecology and geology. Hence, the applications of the generalized models to these fields are clear requisite. The generalization techniques such as either inserting one or more shape parameters or transforming of the parent distribution are useful to (i) increase the applicability of a parent distribution; (ii) explore skewness and tail properties and (iii) improve the goodness-of-fit of the generalized distributions.

A flexible model for the analysis of lifetime data sets is often attractive to the researchers. The moment exponential (ME) distribution was established by Dara and Ahmad [1]. The probability density function (pdf) and cumulative distribution function (cdf) of the ME distribution are given, respectively, by

$$g(x)=\frac{x}{\lambda^{2}}e^{-\frac{x}{\lambda }},x>0,$$ (1)

and

$$G(x)=1-\left(1+\frac{x}{\lambda }\right)e^{-\frac{x}{\lambda }},x\geq 0,$$ (2)

where the r^th^ moment about the origin is

$$\mu_{r}^{\prime }=E(X^{r})=\lambda^{r}\Gamma (2+r).$$

The odds ratio for the ME random variable X is given by

$$W(G(x))=\frac{G(x;\lambda )}{\bar{G}(x;\lambda )}=[\frac{1-(1+\frac{x}{\lambda })e^{-\frac{x}{\lambda }}}{(1+\frac{x}{\lambda })e^{-\frac{x}{\lambda }}}].$$

During the recent years, the ME distribution has been of great interest in literature. Some new extensions of the ME distribution are: exponentiated ME (EME) distribution (Hasnain et al. [2]), generalized exponentiated ME (GEME) distribution (Iqbal et al. [3]), Weibull ME (WME) distribution (Hashmi et al. [4]) and Topp-Leone moment exponential distribution (Abbas et al. [5]). However, new flexible generalizations of the ME distribution are still needed.

The Burr-XII (BXII) distribution among Burr family (Burr [6]) is widely applied to model insurance data and failure time data. Many generalization of the BXII distributions are available in literature such as Burr XII power series (Silva and Cordeiro [7]), Burr XII modified Weibull (Mdlongwa et al. [8]), Burr XII Uniform (Nasir et al. [9]), Burr XII—Weibull (Kyurkchiev et al. [10]), Burr XII system of densities (Cordeiro et al. [11]), New Burr XII-Weibull-logarithmic (Oluyede et al. [12]), Burr XII-exponential (Yari and Tondpour [13]), Burr XII-Burr XII (Gad et al. [14]) and Burr XII inverse Rayleigh (Goual and Yousof [15]).

The idea here is to incorporate the ME distribution into a larger family through an application of the Burr XII (BXII) distribution. In fact, based on the T-X transform defined by Alzaatreh et al. [16], we construct the BXII-ME distribution.

The study is based on the following motivations: (i) to generate distributions with symmetrical, right-skewed, left-skewed, J, reverse-J and bimodal shaped as well as high kurtosis; (ii) to have monotone and non-monotone failure rate function; (iii) to study numerically the descriptive measures for the BXII-ME distribution based on the parameter values; (iv) to derive mathematical properties such as random number generator, sub-models, ordinary moments, conditional moments, reliability measures and characterizations; (v) to perform the simulation study on the basis of the graphical results to see the performance of maximum likelihood, maximum product spacings, least squares, weighted least squares, Cramer-von Mises and Anderson-Darling estimators; (vi) to reveal the potentiality of the BXII-ME model; (vii) to work as the preeminent substitute model and (viii) to deliver a better fit model than the existing models.

The contents of the article are structured as follows. Section 2 derives the BXII-ME model. We study basic structural properties such as random number generator and sub-models for the BXII-ME model. Section 3 presents certain mathematical properties such as the ordinary moments, conditional moments, reliability measures and characterizations. Section 4 addresses six estimation methods to estimate the BXII-ME parameters. In Section 5, we perform simulation studies on the basis of the graphical results to see the performance of maximum likelihood, maximum product spacings, least squares, weighted least squares, Cramer-von Mises and Anderson-Darling estimators of the BXII-ME distribution. In Section 6, we apply the BXII-ME distribution to two real data sets by adopting maximum likelihood estimation method. We also verify the potentiality of the BXII-ME model. In Section 7, we conclude the article.

## 2. The BXII-ME distribution

In this section, we derive the BXII-ME distribution from the T-X family technique. We also obtain the BXII-ME model by linking the exponential and gamma variables. Basic structural properties are studied. Then, we highlight the nature of the density and failure rate functions.

### 2.1 T-X family technique

To obtain a wider family of distributions, Alzaatreh et al. [16] derived the cdf for the T*-X* family as follows:

$$F(x)=\int_{a}^{W[G(x;\xi )]}r(t)dt,\;x\in R,$$ (3)

where *r*(*t*) is the pdf of the random variable *T*, where *T*∈ [*a*, *b*] for −∞≤ *a*<*b* < ∞ and *W*[*G*(*x*;*ξ*)] is a function of the baseline cdf of a random variable *X*, subject to the vector parameter *ξ* and satisfying

- *W*[*G*(*x*; *ξ*)]∈[*a*, *b*],
- *W*[*G*(*x*; *ξ*)] is differentiable and monotonically non-decreasing and
- $\underset{x\to -\infty }{lim}W[G(x;\xi )]\to a\;\mathrm{and}\;\underset{x\to \infty }{lim}W[G(x;\xi )]\to b$.

For the T*-X* family of distributions, the pdf of X is given by

$$f(x)=\left\{\frac{\partial }{\partial x}W\left[G(x;\xi )\right]\right\}r\{W[G(x;\xi )]\},x\in R.$$ (4)

We derive the cdf of the BXII-ME distribution via the T-X family technique by setting

$$r\left(t\right)=\alpha \beta t^{\beta -1}{\left(1+t^{\beta }\right)}^{-\alpha -1},t>0,\alpha >0,\beta >0$$

and

$$W\left(G(x)\right)=\left[\frac{1-\left(1+\frac{x}{\lambda }\right)e^{-\frac{x}{\lambda }}}{\left(1+\frac{x}{\lambda }\right)e^{-\frac{x}{\lambda }}}\right].$$

Then, the cdf of BXII-ME distribution is

$$F(x;\alpha ,\beta ,\lambda )=\int_{0}^{\left[\frac{1-\left(1+\frac{x}{\lambda }\right)e^{-\frac{x}{\lambda }}}{\left(1+\frac{x}{\lambda }\right)e^{-\frac{x}{\lambda }}}\right]}\alpha \beta t^{\beta -1}{\left(1+t^{\beta }\right)}^{-\alpha -1}dt,$$

or

$$F\left(x\right)=1-{\left\{1+{\left[\frac{1-\left(1+\frac{x}{\lambda }\right)e^{-\frac{x}{\lambda }}}{\left(1+\frac{x}{\lambda }\right)e^{-\frac{x}{\lambda }}}\right]}^{\beta }\right\}}^{-\alpha },x\geq 0,$$ (5)

where α, β, λ > 0 are parameters. The pdf corresponding to (5) is given by

$$f\left(x\right)=\alpha \beta \frac{x}{\lambda^{2}}e^{-\frac{x}{\lambda }}\frac{{\left[1-\left(1+\frac{x}{\lambda }\right)e^{-\frac{x}{\lambda }}\right]}^{\beta -1}}{{\left[\left(1+\frac{x}{\lambda }\right)e^{-\frac{x}{\lambda }}\right]}^{\beta +1}}{\left\{1+{\left[\frac{1-\left(1+\frac{x}{\lambda }\right)e^{-\frac{x}{\lambda }}}{\left(1+\frac{x}{\lambda }\right)e^{-\frac{x}{\lambda }}}\right]}^{\beta }\right\}}^{-\alpha -1},x>0.$$ (6)

In future, a random variable (rv) with pdf (6) is denoted by X~BXII-ME (α,β,λ). For α = 1, the BXII-ME distribution reduces to the Log-logistic-ME (LL-ME) and for β = 1, the BXII-ME distribution reduces to the Lomax-ME (L-ME).

### 2.2 Nexus between gamma and exponential variables

We derive the BXII-ME distribution from the nexus between exponential and gamma variables.

**Lemma (i):** Let *W*_1_ and *W*_2_ be independently distributed random variables but have different probability distributions. The random variable *W*_1_ has exponential distribution with parameter value 1, i.e. *W*_1_~exp(1) and *W*_2_ has gamma distribution with parameters *α* and 1, i.e. *W*_2_~gamma (*α*, 1), then for

$$W_{1}={\left[\frac{1-\left(1+\frac{X}{\lambda }\right)e^{-\frac{X}{\lambda }}}{\left(1+\frac{X}{\lambda }\right)e^{-\frac{X}{\lambda }}}\right]}^{\beta }W_{2},\;\mathrm{we}\;\mathrm{reach}\;\mathrm{at}\;X=-\lambda -\lambda W_{-1}{\left[-e-e{\left(\frac{W_{1}}{W_{2}}\right)}^{\frac{1}{\beta }}\right]}^{-1}∼\mathrm{BXII}‐\mathrm{ME}(\alpha ,\beta ,\lambda ).$$

where *W*_−1_[−exp(−*x*)] = −*x* and *W*_−1_ is the second branch of Lambert-W function.

**Proof**

If *W*_1_~exp(1), i.e. $f\left(w_{1}\right)=e^{-w_{1}},w_{1}>0$.

If *W*_2_~*gamma*(α,1), i.e. $f\left(w_{2}\right)=\frac{w_{2}{}^{\alpha -1}e^{-w_{2}}}{\Gamma \left(\alpha \right)},w_{2}>0.$ Then, the joint distribution of two random variables (rvs) is

$$f\left(w_{1},w_{2}\right)=\frac{w_{2}{}^{\alpha -1}e^{-w_{2}}e^{-w_{1}}}{\Gamma \left(\alpha \right)},\;w_{1}>0,\;w_{2}>0.$$

Letting $W_{1}={\left[\frac{1-\left(1+\frac{X}{\lambda }\right)e^{-\frac{X}{\lambda }}}{\left(1+\frac{X}{\lambda }\right)e^{-\frac{X}{\lambda }}}\right]}^{\beta }W_{2}$, the joint density of the rvs *X* and *W*_2_ is

$$f\left(x,w_{2}\right)=\frac{w_{2}{}^{\alpha -1}e^{-w_{2}}e^{-{\left[\frac{1-\left(1+\frac{x}{\lambda }\right)e^{-\frac{x}{\lambda }}}{\left(1+\frac{x}{\lambda }\right)e^{-\frac{x}{\lambda }}}\right]}^{\beta }w_{2}}}{\Gamma \left(\alpha \right)}\beta \frac{x}{\lambda^{2}}e^{-\frac{x}{\lambda }}\frac{{\left[1-\left(1+\frac{x}{\lambda }\right)e^{-\frac{x}{\lambda }}\right]}^{\beta -1}}{{\left[\left(1+\frac{x}{\lambda }\right)e^{-\frac{x}{\lambda }}\right]}^{\beta +1}}w_{2},x>0,w_{2}>0.$$

The BXII-ME density of *X* is

$$f\left(x\right)=\beta \frac{x}{\lambda^{2}}e^{-\frac{x}{\lambda }}\frac{{\left[1-\left(1+\frac{x}{\lambda }\right)e^{-\frac{x}{\lambda }}\right]}^{\beta -1}}{{\left[\left(1+\frac{x}{\lambda }\right)e^{-\frac{x}{\lambda }}\right]}^{\beta +1}}\int_{0}^{\infty }\frac{w_{2}{}^{\alpha }e^{-w_{2}}}{\Gamma \left(\alpha \right)}\exp\left\{-{\left[\frac{1-\left(1+\frac{x}{\lambda }\right)e^{-\frac{x}{\lambda }}}{\left(1+\frac{x}{\lambda }\right)e^{-\frac{x}{\lambda }}}\right]}^{\beta }w_{2}\right\}dw_{2}.$$

After simplifying, we attain at

$$f\left(x\right)=\alpha \beta \frac{x}{\lambda^{2}}e^{-\frac{x}{\lambda }}\frac{{\left[1-\left(1+\frac{x}{\lambda }\right)e^{-\frac{x}{\lambda }}\right]}^{\beta -1}}{{\left[\left(1+\frac{x}{\lambda }\right)e^{-\frac{x}{\lambda }}\right]}^{\beta +1}}{\left\{1+{\left[\frac{1-\left(1+\frac{x}{\lambda }\right)e^{-\frac{x}{\lambda }}}{\left(1+\frac{x}{\lambda }\right)e^{-\frac{x}{\lambda }}}\right]}^{\beta }\right\}}^{-\alpha -1},x>0.$$

Hence the proof is completed.

### 2.3 Basic structural properties

If X~BXII-ME (α,β,λ), the survival, failure rate, cumulative hazard and reverse hazard functions and elasticity of X are given, respectively, by

$$S(x)={\left\{1+{\left[\frac{1-\left(1+\frac{x}{\lambda }\right)e^{-\frac{x}{\lambda }}}{\left(1+\frac{x}{\lambda }\right)e^{-\frac{x}{\lambda }}}\right]}^{\beta }\right\}}^{-\alpha },x\geq 0,$$ (7)

$$h(x)=-\frac{d}{dx}\ln\left({\left\{1+{\left[\frac{1-\left(1+\frac{x}{\lambda }\right)e^{-\frac{x}{\lambda }}}{\left(1+\frac{x}{\lambda }\right)e^{-\frac{x}{\lambda }}}\right]}^{\beta }\right\}}^{-\alpha }\right),\;x>0,$$ (8)

$$H(x)=\alpha \;\ln\left\{1+{\left[\frac{1-\left(1+\frac{x}{\lambda }\right)e^{-\frac{x}{\lambda }}}{\left(1+\frac{x}{\lambda }\right)e^{-\frac{x}{\lambda }}}\right]}^{\beta }\right\}x\geq 0,$$ (9)

$$r(x)=\frac{d\ln}{dx}\left(1-{\left\{1+{\left[\frac{1-\left(1+\frac{x}{\lambda }\right)e^{-\frac{x}{\lambda }}}{\left(1+\frac{x}{\lambda }\right)e^{-\frac{x}{\lambda }}}\right]}^{-\beta }\right\}}^{-\alpha }\right),\;x>0$$ (10)

and

$$\eta_{F}\left(x\right)=\frac{d}{d\ln x}\left(\ln{\left\{1+{\left[\frac{1-\left(1+\frac{x}{\lambda }\right)e^{-\frac{x}{\lambda }}}{\left(1+\frac{x}{\lambda }\right)e^{-\frac{x}{\lambda }}}\right]}^{-\beta }\right\}}^{-\alpha }\right).$$ (11)

The quantile function of the BXII-ME distribution for 0<*q*<1 is

$$x_{q}=-\lambda \left\{1+W_{-1}\left[-\left\{{\left(\left\{1+{\left[{\left(1-q\right)}^{-\frac{1}{\alpha }}-1\right]}^{\frac{1}{\beta }}\right\}e\right)}^{-1}\right\}\right]\right\},$$ (12)

where *W*_−1_ is the second branch of Lambert-W function (Jodra [17]).

Then, the rv

$$X=-\lambda \left\{1+W_{-1}\left[-\left\{{\left(\left\{1+{\left[{\left(1-Z\right)}^{-\frac{1}{\alpha }}-1\right]}^{\frac{1}{\beta }}\right\}e\right)}^{-1}\right\}\right]\right\}.$$ (13)

is the BXII-ME rv, where Z is the uniform rv defined on (0,1) interval.

### 2.4 Shapes of the density and failure rate functions

**Fig 1:**
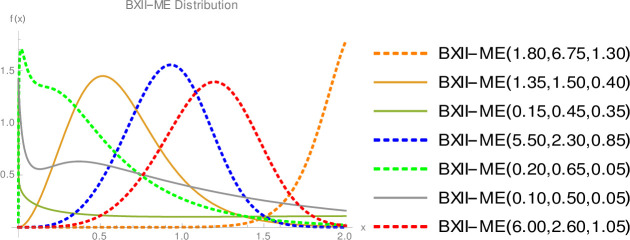
Plots of the BXII-ME density.

**Fig 2:**
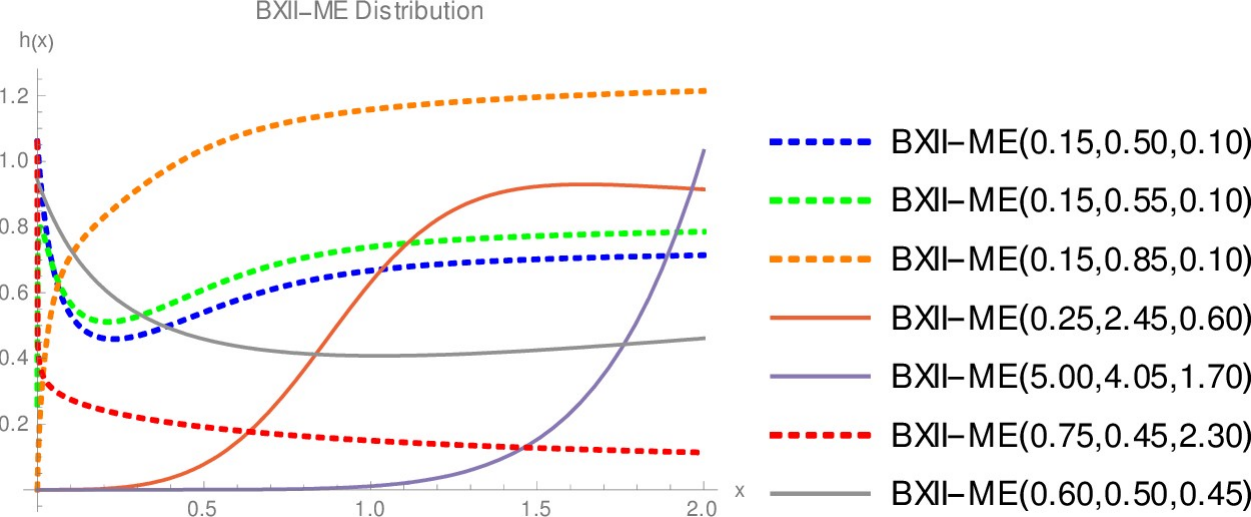
Plots of the BXII-ME hazard rate.

Since we do not decide shapes of the density and hazard rate functions analytically, we plot them based on some selected parameters values to see their possible shapes. Fig 1 displays that BXII-ME density can take various shapes such as symmetrical, right-skewed, left-skewed, J, reverse-J and bimodal. Fig 2 shows that failure rate function can be modified bathtub, bathtub, inverted bathtub, increasing, decreasing, increasing-decreasing and decreasing-increasing-decreasing shaped. Therefore, the BXII-ME distribution is quite flexible and can be applied to numerous data sets.

### 2.5 Linear representation

In this subsection, we provide a useful linear representation for the density of *X*, which can be used to derive some mathematical properties of the BXII-ME model. The cdf (5) can be expressed as

$$F_{\Theta }\left(x\right)=1-\underset{A\left(x\right)}{\underset{︸}{{\left(1+{\left\{\frac{1-\left(1+\frac{x}{\lambda }\right)e^{-\frac{x}{\lambda }}}{1-\left[1-\left(1+\frac{x}{\lambda }\right)e^{-\frac{x}{\lambda }}\right]}\right\}}^{\beta }\right)}^{-\alpha }}}.$$ (14)

First, we shall consider the three power series

$${\left(1+c\right)}^{-\beta }=\overset{\infty }{\underset{\zeta =0}{\sum }}{\left(-1+c\right)}^{\zeta }{\left(\frac{1}{2}\right)}^{\beta +\zeta }\left(\begin{array}{c} -\beta \\ \zeta \end{array}\right),$$ (15)

$${\left(1-c\right)}^{-\beta }=\overset{\infty }{\underset{j=0}{\sum }}\frac{\Gamma \left(\beta +j\right)}{j!\Gamma \left(\beta \right)}c^{j}{|}_{\left(\left|c\right|<1,\beta >0\right)},$$ (16)

and the generalized binomial series given by

$${\left(1-c\right)}^{\eta -1}=\overset{\infty }{\underset{r=0}{\sum }}\frac{{\left(-1\right)}^{r}\Gamma \left(\eta \right)}{r!\Gamma \left(\eta -r\right)}c^{r}{|}_{\left(\left|c\right|<1and\;\eta >0\;real\;non-integer\right)}.$$ (17)

Applying (15) for *A*(*x*) in (14), we obtain

$$F\left(x\right)=1-\sum_{\zeta =0}^{\infty }{\left(\frac{1}{2}\right)}^{\alpha +\zeta }\left({}_{\zeta }^{-\alpha }\right){\left({\left\{\frac{1-\left(1+\frac{x}{\lambda }\right)e^{-\frac{x}{\lambda }}}{1-\left[1-\left(1+\frac{x}{\lambda }\right)e^{-\frac{x}{\lambda }}\right]}\right\}}^{\beta }-1\right)}^{\zeta }.$$

Second, using the binomial expansion, the last equation can be expressed as

$$F\left(x\right)=1-\sum_{\zeta =0}^{\infty }\sum_{i=0}^{\zeta }{\left(-1\right)}^{i}\left({}_{i}^{\zeta }\right)\left({}_{\zeta }^{-\alpha }\right){\left(\frac{1}{2}\right)}^{\alpha +\zeta }{\left[1-\left(1+\frac{x}{\lambda }\right)e^{-\frac{x}{\lambda }}\right]}^{\beta \left(\zeta -i\right)}\underset{B\left(x\right)}{\underset{︸}{{\left\{1-\left[1-\left(1+\frac{x}{\lambda }\right)e^{-\frac{x}{\lambda }}\right]\right\}}^{-\beta \left(\zeta -i\right)}}},$$

Third, applying (16) for *B*(*x*) in the last equation, we can write

$$F\left(x\right)=1-\sum_{,j,\zeta =0}^{\infty }\sum_{i=0}^{\zeta }{\left(-1\right)}^{i}\left({}_{i}^{\zeta }\right)\left({}_{\zeta }^{-\alpha }\right){\left(\frac{1}{2}\right)}^{\alpha +\zeta }\frac{\Gamma \left(\beta^{*}\right)}{j!\Gamma \left[\beta \left(\zeta -i\right)\right]}{\left[1-\left(1+\frac{x}{\lambda }\right)e^{-\frac{x}{\lambda }}\right]}^{\beta^{*}}$$

$$F\left(x\right)=1-\sum_{j,\zeta =0}^{\infty }\sum_{i=0}^{\zeta }d_{i,j,\zeta }\Pi_{\beta^{*}}\left(x\right),$$ (18)

is the cdf of the exponentiated ME (Exp-ME) distribution with the power parameter [β* = *β*(*ζ*−*i*)+*j*]>0, where

$$\Pi_{\beta^{*}}\left(x\right)={\left[1-\left(1+\frac{x}{\lambda }\right)e^{-\frac{x}{\lambda }}\right]}^{\beta^{*}},$$

and

$$d_{i,j,\zeta }={\left(-1\right)}^{i}\left({}_{i}^{\zeta }\right)\left({}_{\zeta }^{-\alpha }\right){\left(\frac{1}{2}\right)}^{\alpha +\zeta }\frac{\Gamma \left(\beta^{*}\right)}{j!\Gamma \left[\beta \left(\zeta -i\right)\right]}.$$

Upon differentiating (18), we obtain

$$f(x)=\sum_{j,\zeta =0}^{\infty }\sum_{i=0}^{\zeta }C_{i,j,\varsigma }\pi_{\beta^{*}}(x),$$ (19)

where

$$C_{i,j,\zeta }={-d}_{i,j,\zeta }$$

and

$$\pi_{\beta^{*}}\left(x\right)=\frac{\beta^{*}}{\lambda^{2}}xe^{-\frac{x}{\lambda }}{\left[1-\left(1+\frac{x}{\lambda }\right)e^{-\frac{x}{\lambda }}\right]}^{\beta^{*}-1}$$

is the density of the Exp-ME model with the power parameter β*. Eq (19) reveals that the BXII-ME is a linear combination of Exp-ME densities.

## 3. Mathematical properties

We present some of its mathematical properties such as the ordinary moments, the Mellin transform, conditional moments, reliability measures and characterization in this segment.

### 3.1 Moments

The moments are significant tools for statistical analysis in pragmatic sciences. The r^th^ moment about the origin is

$$E\left(X^{r}\right)=\int_{0}^{\infty }x^{r}\overset{\infty }{\underset{j,\zeta =0}{\sum }}\overset{\zeta }{\underset{i=0}{\sum }}C_{i,j,\zeta }\frac{\beta^{*}}{\lambda^{2}}xe^{-\frac{x}{\lambda }}{\left[1-\left(1+\frac{x}{\lambda }\right)e^{-\frac{x}{\lambda }}\right]}^{\beta^{*}-1}dx,$$

$$\mu_{r}^{\prime }=E\left(X^{r}\right)=\overset{\infty }{\underset{j,\zeta =0}{\sum }}\overset{\zeta }{\underset{i=0}{\sum }}\sum_{l=0}^{\beta^{*}-1}\sum_{m=0}^{l}C_{i,j,\zeta }{\left(-1\right)}^{l}\left({}_{l}^{\beta^{*}-1}\right)\left({}_{m}^{l}\right)\frac{\beta^{*}\lambda^{r}\Gamma \left[r+m+2\right]}{{\left(l+1\right)}^{r+m+2}},r=1,2,3,\ldots$$ (20)

where Γ[,.] is the gamma function.

The Mellin transformation is applied to obtain the moments as

$$M\left\{f\left(x\right);r\right\}=\int_{0}^{\infty }x^{r-1}\overset{\infty }{\underset{j,\zeta =0}{\sum }}\overset{\zeta }{\underset{i=0}{\sum }}C_{i,j,\zeta }\frac{\beta^{*}}{\lambda^{2}}xe^{-\frac{x}{\lambda }}{\left[1-\left(1+\frac{x}{\lambda }\right)e^{-\frac{x}{\lambda }}\right]}^{\beta^{*}-1}dx,$$

$$Μ\left\{f\left(x\right);r\right\}=\overset{\infty }{\underset{j,\zeta =0}{\sum }}\overset{\zeta }{\underset{i=0}{\sum }}\sum_{l=0}^{\beta^{*}-1}\sum_{m=0}^{l}C_{i,j,\zeta }{\left(-1\right)}^{l}\left({}_{l}^{\beta^{*}-1}\right)\left({}_{m}^{l}\right)\frac{\beta^{*}\lambda^{r-1}\Gamma \left[r+m+1\right]}{{\left(l+1\right)}^{r+m+1}},\;r=1,2,3,\ldots .$$ (21)

The r^th^ central moment (μ_*r*_), skewness (γ_1_) and kurtosis (γ_2_) for the BXII-ME model are obtained from $\mu_{r}=\sum_{l=1}^{r}{\left(-1\right)}^{l}\left({}_{l}^{r}\right){\mu^{\prime }}_{l}{\mu^{\prime }}_{r-l},$ $\gamma_{1}=\frac{\mu_{3}}{{\left(\mu_{2}\right)}^{\frac{3}{2}}}$ and $\beta_{2}=\frac{{}_{\mu_{4}}}{{\left(\mu_{2}\right)}^{2}}$.The numerical values for mean $\left(\mu_{1}^{\prime }\right)$, median $\left(\tilde{\mu }\right)$, dispersion (σ), γ_1_ and γ_2_ for the BXII-ME distribution for selected values of α,β,λ are listed in Table 1.

**Table 1:** Quantities $\mu_{1}^{\prime }$, $\tilde{\mu }$, σ, γ_1_ and γ_2_ of the BXII-ME distribution.

α,β,λ	$\mu_{1}^{\prime }$	$\tilde{\mu }$	σ	γ_1_	γ_2_
0.5,0.5,0.5	2.6776	1.9401	8.6517	74.8778	6089.09
1,0.5,0.5	1.3043	0.8388	1.3588	1.6362	6.3791
2,0.5,0.5	0.6059	0.3354	0.7108	2.0698	8.5074
3,0.5,0.5	0.3737	0.2033	0.4608	2.4150	10.7472
4,0.5,0.5	0.2611	0.1442	0.3276	2.7008	13.4562
5,0.5,0.5	0.1983	0.1119	0.2486	2.8423	14.8636
0.5,1,0.5	1.6534	1.3440	1.2528	1.5988	7.3620
0.5,2,0.5	1.2092	1.0763	0.6183	1.5854	7.3019
0.5,3,0.5	1.0717	0.9936	0.4024	1.5638	7.6506
0.5,4,0.5	1.0064	0.9534	0.2950	1.5012	7.5854
0.5,5,0.5	0.9690	0.9298	0.2314	1.4358	7.3989
0.5,0.5,1	5.1601	3.8796	4.8952	1.5752	6.7218
0.5,0.5,2	10.3192	7.7575	9.7881	1.5745	6.7248
0.5,0.5,3	15.4789	11.6344	14.6839	1.5752	6.7266
0.5,0.5,4	20.6384	15.5133	19.5786	1.5746	6.7190
0.5,0.5,5	25.7992	19.3969	24.4738	1.5746	6.7197
1.7,5,0.65	1.0197	1.0212	0.1502	0.0016	3.6538
2,5,0.65	0.9984	1.0021	0.1426	-0.1139	3.5415
5,2.85,0.5	0.5972	0.6019	0.1350	-0.1462	3.0058
5,2.8,0.5	0.5938	0.5982	0.1366	-0.1322	2.9973
5,2,0.5	0.5219	0.5190	0.1660	0.1593	2.9411
5.5,1.7398,0.5	0.4735	0.4664	0.1712	0.2921	3.0000

### 3.2 Conditional moments

Life expectancy, mean waiting time and inequality measures can be obtained from the incomplete moments.

The r^th^ conditional moment *E*(*X*^*r*^|*X*>*z*) is $E\left(X^{r}|X>z\right)=\frac{1}{S\left(z\right)}\left[\mu_{r}^{\prime }-E_{X\leq z}\left(X^{r}\right)\right].$ The r^th^ lower incomplete moment *E*_*X*≤*z*_(*X*^*r*^) is

$$E_{X\leq z}\left(X^{r}\right)=\int_{0}^{z}x^{r}\overset{\infty }{\underset{j,\zeta =0}{\sum }}\overset{\zeta }{\underset{i=0}{\sum }}C_{i,j,\zeta }\frac{\beta^{*}}{\lambda^{2}}xe^{-\frac{x}{\lambda }}{\left[1-\left(1+\frac{x}{\lambda }\right)e^{-\frac{x}{\lambda }}\right]}^{\beta^{*}-1}dx,$$

$$M_{r}^{\prime }\left(z\right)=E_{X\leq z}\left(X^{r}\right)=\overset{\infty }{\underset{j,\zeta =0}{\sum }}\overset{\zeta }{\underset{i=0}{\sum }}\sum_{l=0}^{\beta^{*}-1}\sum_{m=0}^{l}C_{i,j,\zeta }{\left(-1\right)}^{l}\left({}_{l}^{\beta^{*}-1}\right)\left({}_{m}^{l}\right)\frac{\beta^{*}\lambda^{r}\gamma \left(z;r+m+2\right)}{{\left(l+1\right)}^{r+m+2}},\;r=1,2,3,\ldots$$ (22)

where *γ*(*z*;…) is the lower incomplete gamma function.

The r^th^ conditional moment is

$$E\left(X^{r}|X>z\right)=\frac{1}{S\left(z\right)}\left\{\overset{\infty }{\underset{j,\zeta =0}{\sum }}\overset{\zeta }{\underset{i=0}{\sum }}\sum_{l=0}^{\beta^{*}-1}\sum_{m=0}^{l}C_{i,j,\zeta }{\left(-1\right)}^{l}\left({}_{l}^{\beta^{*}-1}\right)\left({}_{m}^{l}\right)\frac{\beta^{*}\lambda^{r}}{{\left(l+1\right)}^{r+m+2}}\left[\Gamma \left(r+m+2\right)-\gamma \left(z;r+m+2\right)\right]\right\},$$ (23)

where Γ(*z*;…) is the upper incomplete gamma function.

The r^th^ reversed conditional moment *E*(*X*^*r*^|*X*≤*z*) for X~BXII-ME (α,β,λ), is

$$E\left(X^{r}|X\leq z\right)=\frac{1}{F\left(z\right)}\overset{\infty }{\underset{j,\zeta =0}{\sum }}\overset{\zeta }{\underset{i=0}{\sum }}\sum_{l=0}^{\beta^{*}-1}\sum_{m=0}^{l}C_{i,j,\zeta }{\left(-1\right)}^{l}\left({}_{l}^{\beta^{*}-1}\right)\left({}_{m}^{l}\right)\frac{\beta^{*}\lambda^{r}\gamma \left(z;r+m+2\right)}{{\left(l+1\right)}^{r+m+2}},\;r=1,2,3,\ldots .$$ (24)

The mean deviation about the mean (*δ*_1_ = *E*|*X*−*μ*|) and about the median $\left(\delta_{2}=E\left|X-\tilde{\mu }\right|\right)$ can be written as $\delta_{1}=2\mu F\left(\mu \right)-2\mu M_{1}^{\prime }\left(\mu \right)$ and $\delta_{2}=\mu -2M_{1}^{\prime }\left(\tilde{\mu }\right)$, respectively, where *μ* = *E*(*X*) and $\tilde{\mu }=x_{0.5}$. The quantities $M_{1}^{\prime }\left(\mu \right)$ and $M_{1}^{\prime }\left(\tilde{\mu }\right)$ can be obtained from (22). For specific probability p, Lorenz and Bonferroni curves are computed as $L(p)=\frac{M_{1}^{\prime }\left(q\right)}{\mu^{\prime }}$ and B(*p*) = *L*(*p*)|*p*, where *q* = *Q*(*p*).

### 3.3 Reliability estimation of multicomponent stress-strength model

Consider a system with κ identical elements, out of which s elements are operative. Let X_*i*_,*i* = 1,2…κ represent the strengths of κ elements with the cdf *F* while, the stress Y enforced on the elements has the cdf *G*. The strengths *X*_*i*_ and stress Y are independently and identically distributed (i.i.d.). The probability that system operates properly, is the reliability of the system, i.e.

$$R_{s,\kappa }=P[\mathrm{at}\;\mathrm{the}\;\mathrm{minimum}"s"of(X_{i},i=1,2\ldots \kappa )exceed\;Y].$$

Then, we can write this probability (from Bhattacharyya and Johnson [18]) as follows:

$$R_{s,\kappa }=\sum_{l=s}^{\kappa }\left(\begin{array}{l} \kappa \\ l \end{array}\right)\int_{-\infty }^{\infty }{\left[1-F(y)\right]}^{l}{\left[F(y)\right]}^{\kappa -l}dG(y).$$

Let X~BXII-ME (α_1_,β,λ), Y~ BXII-ME (α_2_,β,λ) with unknown α_1_ and α_2_, common β,λ where X and Y are independently distributed. The reliability in multicomponent stress-strength model for the BXII-ME distribution is

$$R_{s,\kappa }=\sum_{l=s}^{\kappa }\left(\begin{array}{l} \kappa \\ l \end{array}\right)\int_{0}^{\infty }{\left({\left\{1+{\left[\frac{1-\left(1+\frac{x}{\lambda }\right)e^{-\frac{x}{\lambda }}}{\left(1+\frac{x}{\lambda }\right)e^{-\frac{x}{\lambda }}}\right]}^{\beta }\right\}}^{-\alpha_{1}}\right)}^{l}{\left(\left(1-{\left\{1+{\left[\frac{1-\left(1+\frac{x}{\lambda }\right)e^{-\frac{x}{\lambda }}}{\left(1+\frac{x}{\lambda }\right)e^{-\frac{x}{\lambda }}}\right]}^{\beta }\right\}}^{-\alpha_{1}}\right)\right)}^{\left(\kappa -l\right)}d{\left\{1+{\left[\frac{1-\left(1+\frac{x}{\lambda }\right)e^{-\frac{x}{\lambda }}}{\left(1+\frac{x}{\lambda }\right)e^{-\frac{x}{\lambda }}}\right]}^{\beta }\right\}}^{-\alpha_{2}}.$$

Letting $u={\left\{1+{\left[\frac{1-\left(1+\frac{x}{\lambda }\right)e^{-\frac{x}{\lambda }}}{\left(1+\frac{x}{\lambda }\right)e^{-\frac{x}{\lambda }}}\right]}^{\beta }\right\}}^{-\alpha_{2}}$, we obtain $R_{s,\kappa }=\sum_{l=s}^{\kappa }\left(\begin{array}{l} \kappa \\ l \end{array}\right)\int_{0}^{1}{\left(u^{v}\right)}^{l}{\left(1-u^{v}\right)}^{\left(\kappa -l\right)}du\;where\;v=\frac{\alpha_{1}}{\alpha_{2}}.$

Let *u*^*v*^ = *w*, we have $R_{s,\kappa }=\sum_{l=s}^{\kappa }\left(\begin{array}{l} \kappa \\ l \end{array}\right)\int_{0}^{1}w^{l}{\left(1-w\right)}^{\left(\kappa -l\right)}\frac{1}{\nu }w^{\frac{1}{\nu }-1}dw$

$$R_{s,\kappa }=\frac{1}{\nu }\sum_{l=s}^{\kappa }\left({}_{l}^{\kappa }\right)B\left(l+\frac{1}{\nu },\kappa -l+1\right),$$ (25)

where *B*(.,.) is the beta function. The probability in (25) is known as the reliability of multicomponent stress-strength model. For *s* = *κ* = 1, the multicomponent stress-strength model reduces to the stress-strength model (Kotz et al. [19]) as

$$R_{1,1}=\mathrm{Pr}(Y<X)=\frac{\alpha_{2}}{\left(\alpha_{1}+\alpha_{2}\right)},\;where\;\alpha_{1}+\alpha_{2}>0.$$

### 3.4 Characterizations via truncated moment of a function of the random variable

Here, we characterize the BXII-ME distribution via relationship between truncated moments of a function of X with another function. This characterization is stable in the sense of weak convergence (Glänzel [20]).

**Proposition 3.4.1:** Let *X*:Ω→(0,∞) be a continuous random variable and let

$g\left(x\right)={\left\{1+{\left[\frac{1-\left(1+\frac{x}{\lambda }\right)e^{-\frac{x}{\lambda }}}{\left(1+\frac{x}{\lambda }\right)e^{-\frac{x}{\lambda }}}\right]}^{\beta }\right\}}^{-1},x>0.$ The pdf of X is (6) if and only if the function *h*(*x*), in Theorem G (Glänzel [8]), has the form $h\left(x\right)=\frac{\alpha }{\alpha +1}{\left\{1+{\left[\frac{1-\left(1+\frac{x}{\lambda }\right)e^{-\frac{x}{\lambda }}}{\left(1+\frac{x}{\lambda }\right)e^{-\frac{x}{\lambda }}}\right]}^{\beta }\right\}}^{-1},\;x>0$.

**Proof** If X has pdf (6), then $\left(1-F\left(x\right)\right)E\left(g\left(X\right)|X\geq x\right)=\frac{\alpha }{\alpha +1}{\left\{1+{\left[\frac{1-\left(1+\frac{x}{\lambda }\right)e^{-\frac{x}{\lambda }}}{\left(1+\frac{x}{\lambda }\right)e^{-\frac{x}{\lambda }}}\right]}^{\beta }\right\}}^{-(\alpha +1)},x>0,$ or

$$E\left(g\left(X\right)|X\geq x\right)=\frac{\alpha }{\alpha +1}{\left\{1+{\left[\frac{1-\left(1+\frac{x}{\lambda }\right)e^{-\frac{x}{\lambda }}}{\left(1+\frac{x}{\lambda }\right)e^{-\frac{x}{\lambda }}}\right]}^{\beta }\right\}}^{-1},x>0,$$

and

$$h\left(x\right)-g\left(x\right)=-\frac{1}{\alpha +1}{\left\{1+{\left[\frac{1-\left(1+\frac{x}{\lambda }\right)e^{-\frac{x}{\lambda }}}{\left(1+\frac{x}{\lambda }\right)e^{-\frac{x}{\lambda }}}\right]}^{\beta }\right\}}^{-1},x>0.$$

Conversely, if *h*(*x*) is given as above, then (for *x*>0)

$$h^{\prime }\left(x\right)=-\frac{\alpha }{\alpha +1}\beta \frac{x}{\lambda^{2}}e^{-\frac{x}{\lambda }}\frac{{\left[1-\left(1+\frac{x}{\lambda }\right)e^{-\frac{x}{\lambda }}\right]}^{\beta -1}}{{\left[\left(1+\frac{x}{\lambda }\right)e^{-\frac{x}{\lambda }}\right]}^{\beta +1}}{\left\{1+{\left[\frac{1-\left(1+\frac{x}{\lambda }\right)e^{-\frac{x}{\lambda }}}{\left(1+\frac{x}{\lambda }\right)e^{-\frac{x}{\lambda }}}\right]}^{\beta }\right\}}^{-2}<0,$$

$$s^{\prime }\left(x\right)=\frac{h^{\prime }\left(x\right)}{h\left(x\right)-g\left(x\right)}=\alpha \beta \frac{x}{\lambda^{2}}e^{-\frac{x}{\lambda }}\frac{{\left[1-\left(1+\frac{x}{\lambda }\right)e^{-\frac{x}{\lambda }}\right]}^{\beta -1}}{{\left[\left(1+\frac{x}{\lambda }\right)e^{-\frac{x}{\lambda }}\right]}^{\beta +1}}{\left\{1+{\left[\frac{1-\left(1+\frac{x}{\lambda }\right)e^{-\frac{x}{\lambda }}}{\left(1+\frac{x}{\lambda }\right)e^{-\frac{x}{\lambda }}}\right]}^{\beta }\right\}}^{-1},$$

$$s\left(x\right)=\ln{\left\{1+{\left[\frac{1-\left(1+\frac{x}{\lambda }\right)e^{-\frac{x}{\lambda }}}{\left(1+\frac{x}{\lambda }\right)e^{-\frac{x}{\lambda }}}\right]}^{\beta }\right\}}^{\alpha },x>0,$$

and

$$e^{-s\left(x\right)}={\left\{1+{\left[\frac{1-\left(1+\frac{x}{\lambda }\right)e^{-\frac{x}{\lambda }}}{\left(1+\frac{x}{\lambda }\right)e^{-\frac{x}{\lambda }}}\right]}^{\beta }\right\}}^{-\alpha },x>0.$$

In view of Theorem G, X has density (6).

#### Corollary 3.4.1

Let *X*:Ω→(0,∞) be a continuous random variable. The pdf of X is (6) if and only if there exist functions *h*(*x*) and *g*(*x*) (defined in Theorem G) satisfying the differential equation

$$s^{\prime }\left(x\right)=\alpha \beta \frac{x}{\lambda^{2}}e^{-\frac{x}{\lambda }}\frac{{\left[1-\left(1+\frac{x}{\lambda }\right)e^{-\frac{x}{\lambda }}\right]}^{\beta -1}}{{\left[\left(1+\frac{x}{\lambda }\right)e^{-\frac{x}{\lambda }}\right]}^{\beta +1}}{\left\{1+{\left[\frac{1-\left(1+\frac{x}{\lambda }\right)e^{-\frac{x}{\lambda }}}{\left(1+\frac{x}{\lambda }\right)e^{-\frac{x}{\lambda }}}\right]}^{\beta }\right\}}^{-1},x>0.$$

#### Remark 3.4.1

The general solution of the differential equation in Corollary 3.4.1 is

$$h\left(x\right)={\left\{1+{\left[\frac{1-\left(1+\frac{x}{\lambda }\right)e^{-\frac{x}{\lambda }}}{\left(1+\frac{x}{\lambda }\right)e^{-\frac{x}{\lambda }}}\right]}^{\beta }\right\}}^{\alpha }\left[-\int \alpha \beta \frac{x}{\lambda^{2}}e^{-\frac{x}{\lambda }}\frac{{\left[1-\left(1+\frac{x}{\lambda }\right)e^{-\frac{x}{\lambda }}\right]}^{\beta -1}}{{\left[\left(1+\frac{x}{\lambda }\right)e^{-\frac{x}{\lambda }}\right]}^{\beta +1}}{\left\{1+{\left[\frac{1-\left(1+\frac{x}{\lambda }\right)e^{-\frac{x}{\lambda }}}{\left(1+\frac{x}{\lambda }\right)e^{-\frac{x}{\lambda }}}\right]}^{\beta }\right\}}^{-\alpha -1}g\left(x\right)dx+D\right],$$

where D is a constant.

## 4. Different estimation methods

In this section, we propose various estimators for estimating the unknown parameters of the BXII-ME distribution. We discuss maximum likelihood, maximum product spacings, least squares, weighted least squares, Cramer-von Mises and Anderson-Darling estimation methods and compare their performances on the basis of simulated sample from the BXII-ME distribution. The details are the followings.

### 4.1 Maximum likelihood estimation

We address parameters estimation using maximum likelihood method. The log-likelihood function for the vector of parameters ξ = (α,β,λ) of the BXII-ME distribution is

$$\begin{array}{l} l=l\left(\xi \right)=n\ln\alpha +n\ln\beta -2n\ln\lambda +\sum_{i=1}^{n}\ln x_{i}-\frac{1}{\lambda }\sum x_{i}+\left(\beta -1\right)\sum_{i=1}^{n}\ln\left[1-\left(1+\frac{x_{i}}{\lambda }\right)e^{-\frac{x_{i}}{\lambda }}\right]-\\ \;\left(\beta +1\right)\sum_{i=1}^{n}\ln\left[\left(1+\frac{x_{i}}{\lambda }\right)e^{-\frac{x_{i}}{\lambda }}\right]-\left(\alpha +1\right)\sum_{i=1}^{n}\ln\left\{1+{\left[\frac{1-\left(1+\frac{x_{i}}{\lambda }\right)e^{-\frac{x_{i}}{\lambda }}}{\left(1+\frac{x_{i}}{\lambda }\right)e^{-\frac{x_{i}}{\lambda }}}\right]}^{\beta }\right\}. \end{array}$$ (26)

We can compute the maximum likelihood estimators (MLEs) of α,β and λ by solving equations $\frac{\partial l}{\partial \alpha }=0$, $\frac{\partial l}{\partial \beta }=0$ and $\frac{\partial l}{\partial \lambda }=0$.

### 4.2. Maximum product spacing estimates

The maximum product spacing (MPS) method is alternative method for MLE for parameter estimation. This method was proposed by Cheng and Amin [21,22] as well as it was also independently developed by Ranneby [23] as approximation to the Kullback-Leibler measure of information. This method is based on an idea that differences (spacings) between the values of the cdf at consecutive data points should be identically distributed. Let *X*_(1)_,*X*_(2)_,…,*X*_(*n*)_ be ordered sample of size *n* from BXII-ME distribution. The geometric mean of the differences is given as

$$GM=\sqrt[{n+1}]{\prod_{i=1}^{n+1}D_{i}},$$

where, the difference *D*_*i*_ is defined as

$$D_{i}=\int_{x_{\left(i-1\right)}}^{x_{\left(i\right)}}f\left(x\right)dx;i=1,2,\ldots ,n+1.$$ (27)

The maximum product spacing (MPS) estimates, say ${\hat{\alpha }}_{MPS},{\hat{\beta }}_{MPS}$ and ${\hat{\lambda }}_{MPS}$, of α,β and λ are obtained by maximizing the geometric mean of the differences. Substituting cdf of BXII-ME distribution in Eq (27) and taking logarithm of the above expression, we have

$$MPS\left(\xi \right)=\frac{1}{n+1}\sum_{i=1}^{n+1}\log\left[F\left(x_{\left(i\right)}\right)-F\left(x_{\left(i-1\right)}\right)\right],i=1,2,\ldots ,n+1,$$ (28)

where, *F*(*x*_(0)_) = 0 and *F*(*x*_(*n*+1)_) = 1. The MPSEs ${\hat{\alpha }}_{MPS},{\hat{\beta }}_{MPS}$ and ${\hat{\lambda }}_{MPS}$ are obtained by maximizing *MPS*(ξ).

### 4.3 Least squares estimates

Let *X*_(1)_,*X*_(2)_,…,*X*_(*n*)_ be ordered sample of size n from BXII-ME distribution. Then, the expectation of the empirical cumulative distribution function is defined as

$$E\left[F\left(x_{\left(i\right)}\right)\right]=\frac{i}{n+1};i=1,2,\ldots ,n.$$

The least square estimates (LSEs) say, ${\hat{\alpha }}_{LSE},{\hat{\beta }}_{LSE}$ and ${\hat{\lambda }}_{LSE}$ of α,β and λ are obtained by minimizing

$$QLSE\left(\xi \right)=\sum_{i=1}^{n}{\left(F\left(x_{\left(i\right)}\right)-\frac{i}{n+1}\right)}^{2}.$$ (29)

### 4.4 Weighted least squares estimates

Let *X*_(1)_,*X*_(2)_,…,*X*_(*n*)_ be ordered sample of size n from BXII-ME distribution. The variance of the empirical cumulative distribution function is defined as

$$V\left[F\left(x_{\left(i\right)}\right)\right]=\frac{i(n-i+1)}{(n+2){\left(n+1\right)}^{2}};i=1,2,\ldots ,n.$$

Then, the weighted least square estimates (WLSEs) say, ${\hat{\alpha }}_{\mathrm{WLSE}},{\hat{\beta }}_{WLSE}$ and ${\hat{\lambda }}_{WLSE}$ of α,β and λ are obtained by minimizing

$$QWLSE\left(\xi \right)=\sum_{i=1}^{n}\frac{{\left(F\left(x_{\left(i\right)}\right)-\frac{i}{n+1}\right)}^{2}}{V\left[F\left(x_{\left(i\right)}\right)\right]}.$$ (30)

### 4.5 Anderson-Darling estimation

This estimator is based on Anderson-Darling goodness-of-fits statistics which was introduced by Anderson and Darling [24]. The Anderson-Darling (AD) minimum distance estimates, ${\hat{\alpha }}_{\mathrm{AD}},{\hat{\beta }}_{\mathrm{AD}}$ and ${\hat{\lambda }}_{AD}$ of α,β and λ are obtained by minimizing

$$AD\left(\xi \right)=-n-\sum_{i=1}^{n}\frac{2i-1}{n}\left[\log F\left(x_{\left(i\right)}\right)+\log\left\{1-F\left(x_{\left(n+1-i\right)}\right)\right\}\right].$$ (31)

### 4.6 The Cramer-von Mises estimations

The Cramer-von Mises (CVM) minimum distance estimates, ${\hat{\alpha }}_{\mathrm{CVM}},{\hat{\beta }}_{\mathrm{CVM}}$ and ${\hat{\lambda }}_{\mathrm{CVM}}$, of α,β and λ are obtained by minimizing

$$CVM\left(\xi \right)=\frac{1}{12n}+\sum_{i=1}^{n}{\left[F\left(x_{\left(i\right)}\right)-\frac{2i-1}{2n}\right]}^{2}.$$ (32)

We refer the interested readers to Chen and Balakrishnan [25] for AD and CVM goodness-of-fits statistics. To solve the above equations, Eqs (26) and (28)–(32) can be optimized either directly by using the R (optim and maxLik functions), SAS (PROC NLMIXED) and Ox package (sub-routine Max BFGS) or employing non-linear optimization tactics such as the quasi-Newton procedure to numerically optimize l(ξ), *MPS*(ξ), *QLSE*(ξ), *QWLSE*(ξ), *AD*(ξ) and *CVM*(ξ) functions.

## 5. Simulation experiments

**Fig 3:**
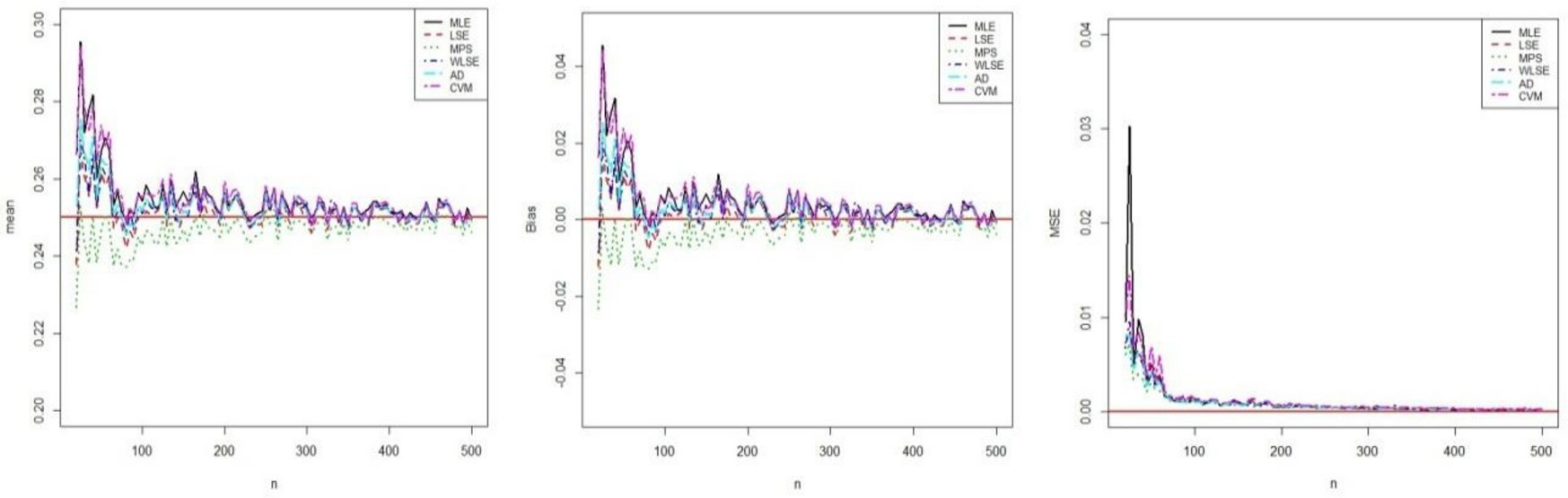
Simulation results of *α*.

**Fig 4:**
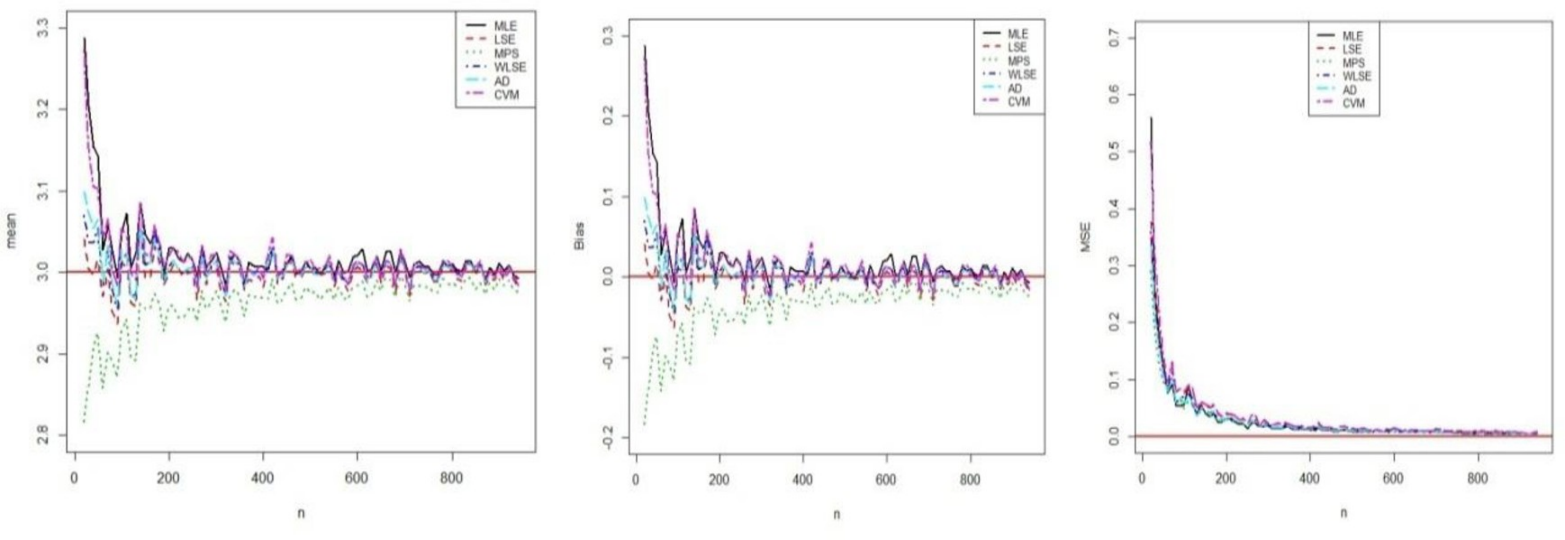
Simulation results of *β*.

**Fig 5:**
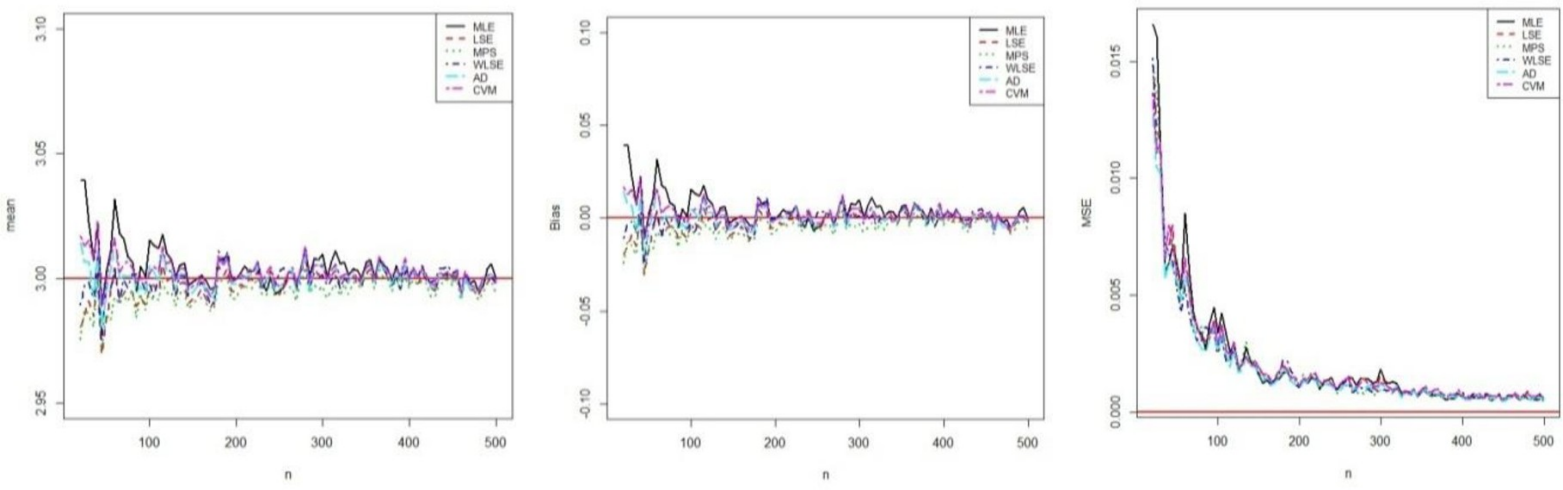
Simulation results of *λ*.

In this Section, we perform the simulation studies by using the BXII-ME to see the performance of the above estimators corresponding to this distribution and obtain the graphical results. We generate N = 1000 samples of size n = 20, 25,…, 500 from BXII-ME distribution with true parameter values α = 0.25,β = 3 and λ = 3. The random numbers generation is obtained by its quantile function. In this simulation study, we calculate the empirical mean, bias and mean square errors (MSEs) of all estimators to compare in terms of their biases and MSEs with varying sample size. The empirical bias and MSE are calculated by (for *h* = *α*,*β*,*λ*)

$$\hat{Bias_{h}}=\frac{1}{N}\sum_{i=1}^{N}\left({\hat{h}}_{i}-h\right),$$

and

$$\hat{MSE_{h}}=\frac{1}{N}\sum_{i=1}^{N}{\left({\hat{h}}_{i}-h\right)}^{2}$$

respectively. All results related to estimations were obtained using optim-CG routine in the R programme. The results of this simulation study are shown in figures (**Figs 3**–**5)**. These Figures show that all estimators are to be consistent since the MSE and biasedness decrease with increasing sample size as expected. It is clear that the estimates of parameters are asymptotically unbiased. For all parameters estimations, the performances of all estimators are close.

## 6. Data applications

We verify the potentiality of the BXII-ME model via monthly actual taxes revenue and fatigue life data sets. The first data set represents monthly actual taxes revenue (in 1000 million Egyptian pounds) from January 2006 to November 2010. It is studied by Nassar and Nada [26] and Yousof et al. [27]. The first data set is available at https://doi.org/10.1080/03610918.2017.1377241. The second data set is about the fatigue life of 6061-T6 aluminum coupons cut parallel with the direction of rolling and oscillated at 18 cycles per second (Birnbaum and Saunders [28] and El-Morshedy et al. [29]). The second data set is available at https://doi.org/10.2307/3212004. We compare the BXII-ME distribution with competing models such as Burr III-moment exponential (BIII-ME), Weibull-moment exponential (W-ME), generalized exponentiated moment exponential (GEME), generalized moment exponential (GME), exponentiated moment exponential (EME), moment exponential (ME) and BXII distributions. For the selection of the best fit distribution, we compute the estimate of likelihood ratio statistics ($-2\overset{⌢}{l}$), Akaike information criterion (AIC), corrected Akaike information criterion (CAIC), Bayesian information criterion (BIC), Hannan-Quinn information criterion (HQIC), Cramer-von Mises (W*), Anderson Darling (A*), and Kolmogorov-Smirnov [K-S] statistics with p-values for all competing models. We compute the MLEs and their standard errors (SEs) in parentheses. Chen and Balakrishnan [25] described in detail about the statistics W* and A*. Chen et al. [30] also studied $-2\overset{⌢}{l}$, AIC and BIC. We also compute the MLEs along with their standard errors (SEs) in parentheses. Table 2 reports some descriptive measures for two real data sets.

**Table 2:** Descriptive statistics.

	N	Min	Max	Mean	Median	Standard deviation	Skewness	Kurtosis
**Monthly Actual Taxes revenue**	59	4.1	39.2	13.4881	10.6	8.0515	1.6083	5.2560
**Fatigue Life**	101	70	212	133.7327	133	22.35571	0.3305	4.05284

Table 2 shows that the monthly actual taxes revenue data set is significantly right-skewed, with significantly positive kurtosis. About the fatigue life data set, it is a right-skewed, with high positive kurtosis.

**Fig 6:**
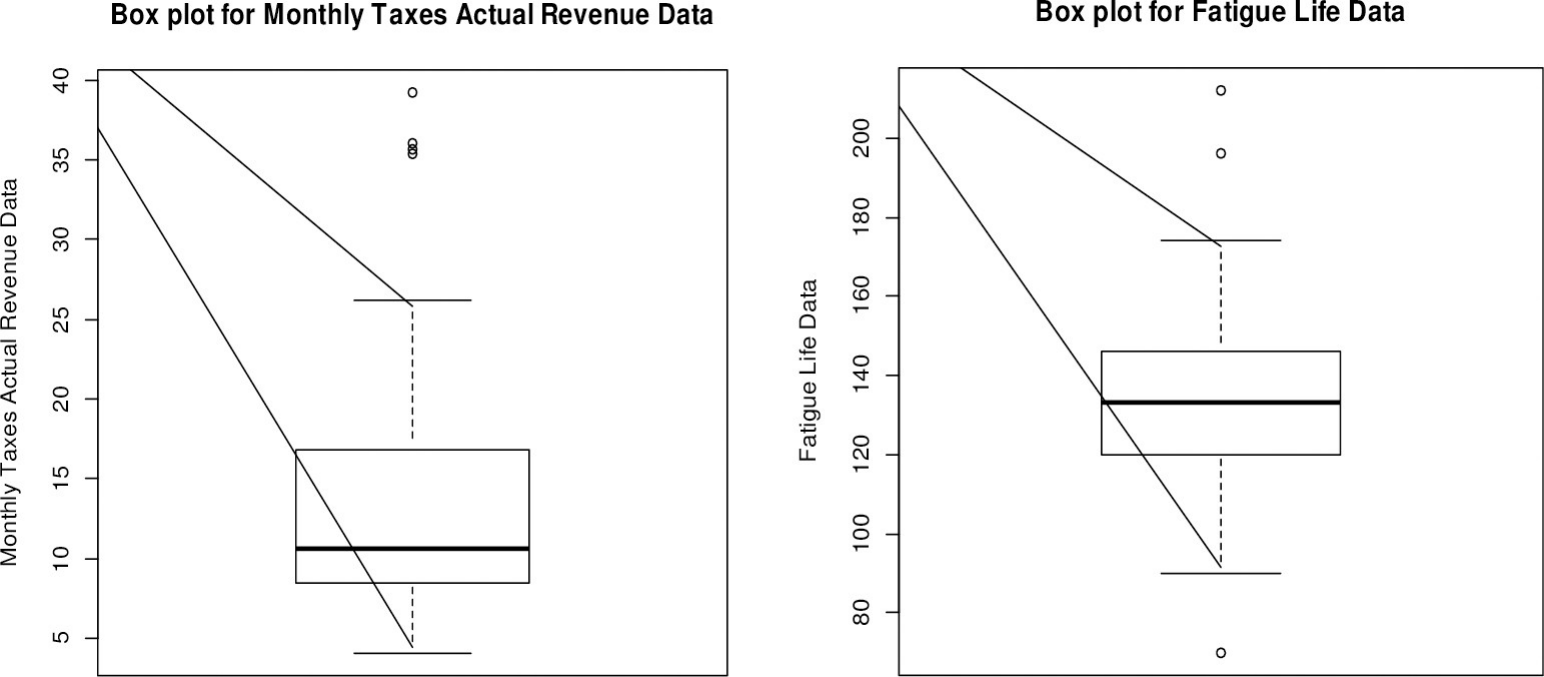
Boxplots of the monthly actual taxes revenue (left) and Fatigue Life (right).

The boxplots in Fig 6 suggests that both data sets are right-skewed. The nature of the two data sets differs in numerous features. Some extreme points are also present in these data sets.

**Fig 7:**
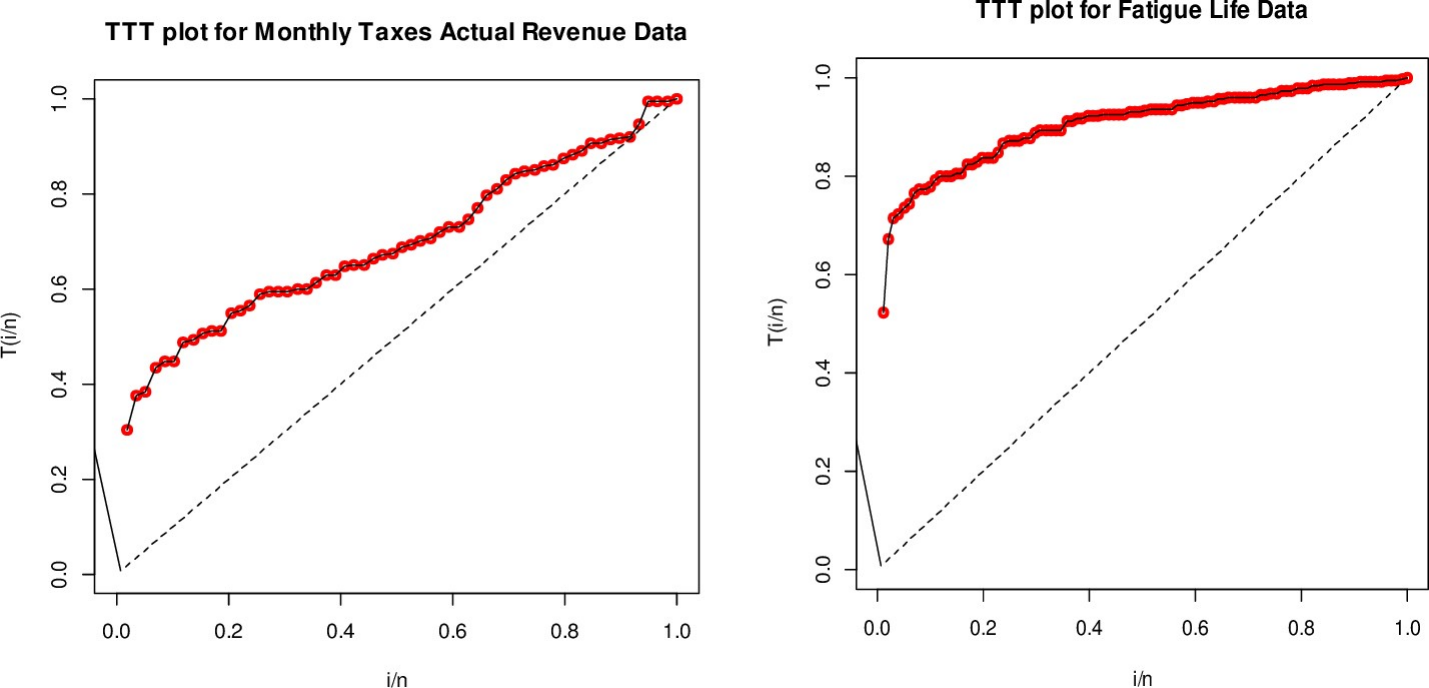
TTT plots of the monthly actual taxes revenue (left) and fatigue life (right).

Here, we study the statistical analysis by total time on test (TTT) for the two data sets in Fig 7.

The TTT plots in Fig 7 for both data sets are concave which suggests increasing failure intensity. So, the BXII-ME distribution is suitable to model these data sets.

### Data set I: Taxes revenue

Table 3 reports the MLEs, SEs and measures W*, A*, K-S (p-values). Table 4 displays the values of measures $-2\overset{⌢}{l}$, AIC, CAIC, BIC and HQIC.

**Table 3:** MLEs, SEs, and W*, A*, K-S (p-values) for monthly actual taxes revenue.

Model	*α*	*β*	*λ*	W*	A*	K-S (p-value)
BXII-ME	0.1601 (0.0724)	3.4311 (1.0493)	3.8680 (0.4363)	0.0304	0.2134	0.0575 (0.9899)
BIII-ME	1.8961 (0.8784)	1.1364 (0.2599)	5.2090 (1.3601)	0.1606	0.9766	0.1206 (0.3572)
W-ME	31.4145 (60.7167)	0.9787 (0.0961)	58.3359 (63.6252)	0.2787	1.7754	0.1409 (0.1918)
GEME	160.3036 (341.4872)	0.3430 (0.1233)	0.3016 (0.1854)	0.05225	0.3049	0.0637 (0.9702)
EME	2.3384 (0.5638)	---	4.5651 (0.5554)	0.1659	1.01409	0.1251 (0.3146)
GME	---	1.3023 (0.1188)	15.7102 (5.5018)	0.2288	1.4390	0.1346 (0.2351)
ME	---	---	6.7441 (0.6208)	0.1949	1.2113	0.1675 (0.07304)
BXII	0.0669 (0.2672)	6.08052 (24.2926)	---	0.05601	0.3254	0.4674 (1.269e-11)

**Table 4:** $-2\overset{⌢}{l}$ AIC, CAIC, BIC and HQIC for monthly actual taxes revenue.

Model	$-2\overset{⌢}{l}$	AIC	CAIC	BIC	HQIC
BXII-ME	375.2900	381.2900	381.7264	387.5227	383.7230
BIII-ME	383.7592	389.7593	390.1956	395.9919	392.1922
W-ME	393.4770	399.4769	399.9133	405.7096	401.9099
GEME	376.6902	382.6901	383.1265	388.9228	385.1231
EME	384.0488	388.0488	388.2630	392.2038	389.6707
GME	389.1094	393.1094	393.3237	397.2645	394.7314
ME	396.1928	398.1928	398.2629	400.2703	399.0038
BXII	514.4602	518.4602	518.6745	522.6153	520.0822

**Fig 8:**
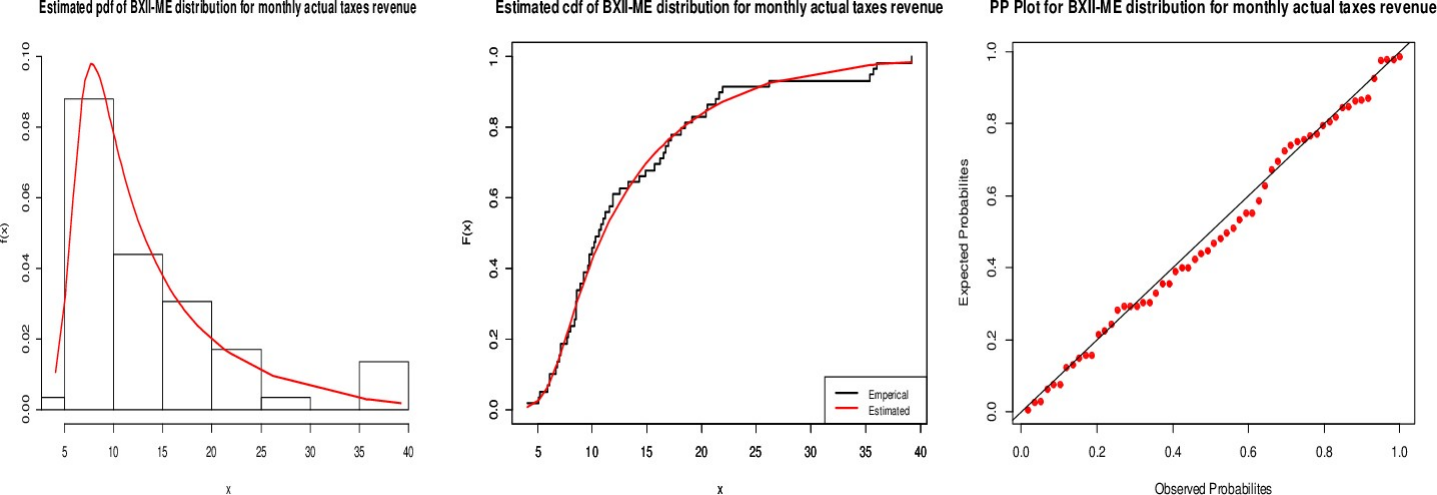
Fitted pdf (left), cdf (center) and PP(right) plots for the BXII-ME distribution to monthly actual taxes revenue.

From the Tables 3 and 4, it is clear that our proposed model is the best fitted, with the smallest values for all statistics and maximum p-value. Fig 8 suggests that the proposed model is closely fitted to monthly actual taxes revenue.

### Data set II: Fatigue life

Table 5 reports the MLEs, SEs (in parentheses) and measures W*, A*, K-S (p-values). Table 6 displays the values of measures $-2\overset{⌢}{l}$, AIC, CAIC, BIC and HQIC.

**Table 5:** MLEs, SEs and W*, A*, K-S (p-values) for fatigue life.

Model	α	β	λ	W*	A*	K-S (p-value)
BXII-ME	1.2646 (0.5203)	4.7614 (0.6304)	81.8650 (5.1685)	**0.0368**	**0.2454**	**0.0524 (0.9441)**
W-ME	52.5287 (90.9450)	3.2035 (0.2283)	160.4654 (43.7269)	0.1521	0.9923	0.1013 (0.2512)
EME	91.2087 (31.4517)	---	18.8163 (1.2031)	0.1836	1.0314	0.1055 (0.211)
GEME	35.3444 (19.9402)	1.2562 (0.1588)	77.9056 (68.0432)	0.1476	0.8293	0.1009 (0.2557)
GME	---	2.9483 (0.0141)	985384.46113 (8451.613)	0.0571	0.3804	0.1597 (0.01156)
EME	---	---	66.86634 (4.7047)	0.0625	0.3795	0.4006 (1.665e-14)
BXII	0.0813 (0.2435)	2.5205 (7.5487)	---	0.0985	0.5657	0.5923 (< 2.2e-16)

**Table 6:** $-2\overset{⌢}{l}$ AIC, CAIC, BIC and HQIC for fatigue life.

Model	$-2\overset{⌢}{l}$	AIC	CAIC	BIC	HQIC
BXII-ME	455.128	916.2559	916.5034	924.1013	919.432
W-ME	463.0179	932.0358	932.2832	939.8812	935.2118
EME	461.6561	927.3123	927.4347	932.5425	929.4296
GEME	459.7397	925.4793	925.7267	933.3247	928.6554
GME	469.9547	943.9095	944.0319	949.1397	946.0268
EME	557.8864	1117.773	1117.813	1120.388	1118.832
BXII	754.1948	1512.390	1512.512	1517.620	1514.507

**Fig 9:**
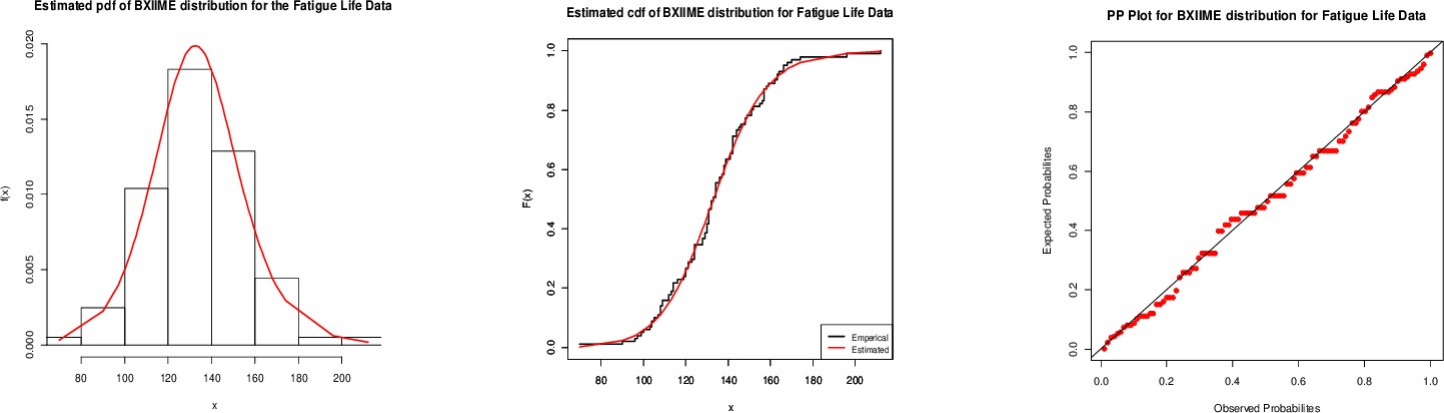
Fitted (left) pdf, (center) cdf and (right) PP plots for the BXII-ME distribution to fatigue life.

From the Tables 5 and 6, it is clear that our proposed model is the best fitted, with the smallest values for all statistics and maximum p-value. Fig 9 infers that the proposed model is closely fitted to fatigue life data.

## 7. Conclusions

We construct the BXII-ME distribution from the T-X family technique. The BXII-ME density highlights various shapes as symmetrical, right-skewed, J, reverse-J, left-skewed, arc and exponential shapes. The BXII-ME failure rate has shapes such as an upside-down bathtub, modified bathtub, constant, increasing, decreasing and increasing-decreasing. We study some of its mathematical properties such as random number generator, sub-models, ordinary moments, conditional moments, reliability measures and characterizations. We employ different estimation methods to estimate the model parameters. We perform simulation studies on the basis of the graphical results to see the performances of the estimators of the BXII-ME distribution. We apply the BXII-ME distribution to two real data sets by adopting maximum likelihood estimation method. The potentiality of the BXII-ME model illustrates that it is flexible, competitive and parsimonious. Therefore it should be included in the distribution theory to facilitate the researchers. Further, as perspective of future projects, we may study some rigorous issues (i) Burr XII generalized moment exponential (BXII-GME) (ii) Burr XII exponentiated moment exponential (BXII- EME); (iii) exponentiated BXII-ME; (iv) unit BXII-ME; (v) bivariate extension of BXII-ME and (vi) discrete case of Burr XII-ME distribution. Future works also includes study of the complexity of the BXII-ME distribution via Bayesian methods. In Bayesian inference, researchers can consider the deviance information criterion (DIC). In this regard, we refer to the articles of [30–35]. We also leave the study of DIC as future work.

## Appendix A

**Theorem G.** Let (Ω,F,P) be a given probability space and let H = [*a*_1_,*a*_2_] be an interval with *a*_1_<*a*_2_ (*a*_1_ = −∞,*a*_2_ = ∞). Let *X*:Ω→[*a*_1_,*a*_2_] be a continuous random variable with distribution function F and Let *g*(*x*) be a real function defined on *H* = [*a*_1_,*a*_2_] such that *E*[*g*(*X*)|X≥x] = *h*(*x)* for *x*∈*H* is defined with some real function *h*(*x*) should be in simple form. Assume that *g*(*x*)ε*C*([*a*_1_,*a*_2_]), *h*(*x*)ε*C*^2^([*a*_1_,*a*_2_]) and F is twofold continuously differentiable and strictly monotone function on the set [*a*_1_,*a*_2_].We conclude, assuming that the equation *g*(*x*) = *h*(*x*) has no real solution in the inside of [*a*_1_,*a*_2_].Then F is obtained from the functions *g*(*x*) and *h*(*x*) as $F\left(x\right)=\int_{a}^{x}k\left|\frac{h^{\prime }\left(t\right)}{h\left(t\right)-g\left(t\right)}\right|\exp\left(-s\left(t\right)\right)dt$, where *s*(*t*) is the solution of equation $s^{\prime }\left(t\right)=\frac{h^{\prime }\left(t\right)}{h\left(t\right)-g\left(t\right)}$ and k is a constant, chosen to make $\int_{a_{1}}^{a_{2}}dF=1.$
